# Identifying Recruitment Sources Across Trophic Levels in a Large River Food Web

**DOI:** 10.1002/ece3.71208

**Published:** 2025-04-03

**Authors:** Shaley A. Valentine, Kristen L. Bouska, Gregory W. Whitledge

**Affiliations:** ^1^ Department of Evolution, Ecology, and Organismal Biology The Aquatic Ecology Laboratory, The Ohio State University Columbus Ohio USA; ^2^ Center for Fisheries, Aquaculture, and Aquatic Sciences and The School of Biological Sciences Southern Illinois University‐Carbondale Carbondale Illinois USA; ^3^ Upper Midwest Environmental Sciences Center U.S. Geological Survey La Crosse Wisconsin USA

**Keywords:** assemblage, environmental history, natal origin, predator–prey, trace element analysis

## Abstract

Assemblages are connected through the movement of physical and biological resources including recruits. Identifying recruitment sources for predators and their prey could help us understand how assemblages use connectivity across multiple trophic levels and whether predator and prey recruitment is coupled. Recruitment sources of organisms across multiple trophic levels can be quantified by trace element analysis of stomach contents. We used trace element analysis of otoliths to determine recruitment contributions from tributaries of predatory largemouth bass ( *Micropterus salmoides* ) and bowfin ( *Amia calva* ) and their consumed prey collected from Pools 4, 8, and 13 of the Upper Mississippi River. We used laser ablation inductively coupled mass spectrometry to quantify strontium:calcium of the core of each otolith and classified each fish to a natal origin (i.e., tributary or potential resident). We compared patterns of natal origin across study reaches, collection years, and species and with previously published origins of independently sampled prey fish. Predator and prey assemblages across all study reaches recruited from tributaries. More prey (44%) than predators (17%) recruited from tributaries. Of fishes originating from tributaries, individuals recruited from various rivers including the large Minnesota and Wisconsin Rivers and several small tributaries. Patterns in natal origin were similar among predators and prey families and among reaches, across sampling years, and between consumed prey and independently sampled prey. Tributaries consistently contributed recruits to both prey and predator fishes, leading to a coupling of predator and prey recruitment sources across space and time. Predators directly and indirectly used tributaries for recruitment and persistence through their own and their prey's recruitment. We further highlighted the utility of using consumed prey to simultaneously study the ecology of prey and predator assemblages, thereby reducing research sampling needs.

## Graphical abstract

We quantified the recruitment sources of predators and their consumed prey across space and time in the Upper Mississippi River. Prey recruited more often than predators from tributaries to the mainstem river, indicating a direct (predators themselves) and indirect (consumed prey) use of tributaries to sustain predator populations. Use of tributaries indicated predator and prey coupling among recruitment sources and was consistent spatially and temporally, suggesting processes leading to these recruitment patterns lead to persistence of species in multiple trophic levels.

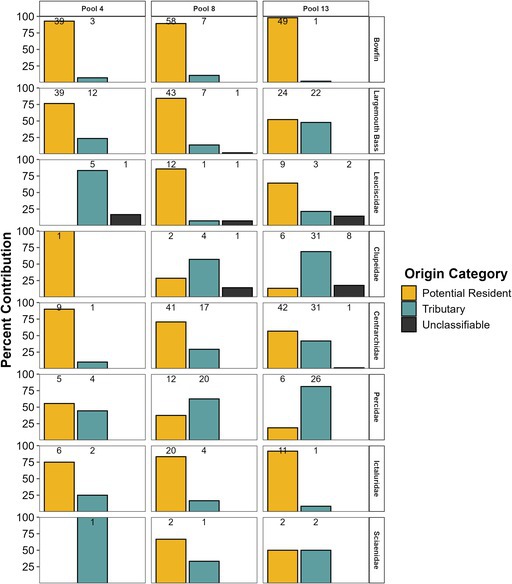

## 1 Introduction

Communities are connected through both physical (e.g., habitat) and biological (e.g., food web) resources. The availability of resources can vary spatiotemporally, and the ability of organisms to obtain resources that meet life history requirements governs community stability (Schlosser 1991; Fausch et al. 2002). To fulfill life history requirements across ontogeny, individuals may rely on connectivity among habitats (e.g., tributaries, mainstem rivers, floodplains, and backwaters) to obtain prey from heterogeneous resource patches (Schlosser 1991; Fausch et al. 2002). However, the scarcity of or unconnected resource patches impedes organisms from gaining necessary resources, and habitat modifications alter the availability of and connectivity among resource patches (Zhang and Xiang 2017), negatively affecting community persistence.

In rivers, subsidies may alleviate alterations to habitat connectivity and subsequent resource use. In situations where autochthonous resources cannot support a community, resources that move across habitat and ecosystem boundaries (i.e., allochthonous resources) can provide sufficient energy and promote community resilience (Nakano and Murakami 2001; Richardson et al. 2010; Zhang and Xiang 2017), alleviating the issue of insufficient resources (Polis et al. 1997; Richardson et al. 2010; Collins et al. 2016). As a specific example, tributaries provide subsidies to mainstem rivers (Rice et al. 2008; Pracheil et al. 2009; Sabo et al. 2018) including physical habitat (Pracheil et al. 2013, 2019), prey subsidies (Rice et al. 2008; Sabo et al. 2018), and recruits (Pracheil et al. 2009; Laughlin et al. 2016; Valentine, Bouska, et al. 2024), regardless of size (Tavernini and Richardson 2020). In modified rivers, tributaries may provide necessary habitat for fish spawning when habitats are unavailable in the mainstem and for life‐long use (Pracheil et al. 2013, 2019; Wohl 2017). Thus, river system connectivity among tributaries and the mainstem river may provide critical resources to mainstem river communities (Bouska et al. 2023).

Identifying the role mainstem river‐tributary connectivity plays in both predator and prey recruitment, and dispersal can inform how habitat use affects the stability of trophic levels. In connected river systems, multiple recruitment (e.g., multiple tributaries and mainstem river habitats) sources can improve the stability of populations (i.e., Portfolio effect). Across time, the identity of those recruitment sources may shift based on environmental perturbations or stochasticity, even when recruitment remains consistent (Brennan and Schindler 2017; Harrison et al. 2020). Further, synchrony in temporal abundance and recruitment of prey and predators can increase ecosystem stability and may be common in at least marine and terrestrial systems (i.e., match‐mismatch hypothesis; White 2007). When combined, intermediate dispersal from spatially heterogenous (Pettersson and Jacobi 2021) and temporally synchronous (Townsend and Gouhier 2019) recruitment sources through habitat connectivity leads to high spatial or temporal coupling in prey and predator abundances, at least in early life stages (e.g., larval). In large rivers, little information exists on the coupling of predator and prey recruitment sources and the role habitat connectivity contributes, especially past the early life stages of organisms. Because predators rely on their own recruitment sources and the recruitment sources of their prey for persistence, identifying where both predators and prey recruit from may yield insights into both recruitment coupling and portfolio effects. Specifically, if predator and prey recruitment sources are similar, predators both directly (themselves) and indirectly (their prey) rely on the same recruitment sources for persistence. Yet, if predator and prey recruitment sources differ, predators directly rely on their own recruitment habitats and indirectly rely on the recruitment habitats of their prey through trophic interactions, indicating a larger portfolio of habitats sustains predators than their own recruitment sources indicate. Therefore, understanding where organisms recruit from across multiple trophic levels and whether multiple habitats act as recruitment sources can yield insights into the degree of habitat connectivity used and the breadth of habitats that sustain assemblages.

Trace element analysis can retroactively identify recruitment sources that contribute to the persistence, stability, and resilience of communities (Pracheil et al. 2009; Laughlin et al. 2016). Trace element analysis of hard parts (e.g., fish otoliths) can provide an environmental history profile of individuals, quantifying previous habitat use across ontogeny (Pracheil et al. 2014). Fish otoliths remain metabolically inert and continuously grow, providing a chronological account of the environments an organism inhabited, allowing for the retroactive determination of used habitats (Campana and Thorrold 2001). Trace element analysis has been used to classify the environmental history of fishes in many aquatic systems (Allen et al. 2009; Pracheil et al. 2014; Daugherty et al. 2019; Rude and Whitledge 2019; Valentine, Bouska, et al. 2024). However, few freshwater studies have quantified the habitat use of small‐bodied or prey fishes (Walther and Thorrold 2008; Radigan et al. 2018; Vivancos et al. 2021; Valentine, Bouska, et al. 2024) and none, to our knowledge, have quantified the environmental history of predators and their consumed prey. Identifying recruitment sources across multiple trophic levels could yield insights into whether the habitat use of predators and prey are coupled and the breadth of the portfolio of habitats lending direct (i.e., recruitment sources) and indirect (i.e., trophic interactions) support to the persistence of assemblages.

Evidence indicates we can identify recruitment sources across trophic levels by quantifying the environmental history of predators and prey in their stomach contents (i.e., Russian doll technique; Grey et al. 2002). Previous marine studies determined recruitment sources of fishes found in the stomachs of predators using stable isotopes or trace element analysis of otoliths (Lenanton et al. 2003; Kemp et al. 2011). Environmental history was successfully determined because digestive processes have little effect on otolith isotopic or trace element ratios as long as otoliths remain intact (Lenanton et al. 2003; Phelps et al. 2009). Thus, the identification of the habitats that support both predator and prey fish recruitment and predator production could be determined using this technique. We currently do not know if recruitment sources between consumed and independently sampled prey are similar. If recruitment sources are comparable, this technique would reduce the need to independently sample predators and prey, thereby reducing the number of samples required (Lenanton et al. 2003; Phelps et al. 2009) and allowing for the study of difficult‐to‐capture or rare prey species if they have been consumed.

The goals of this study were to identify and determine the breadth of recruitment sources that support organisms across multiple trophic levels and determine if using stomach contents is a viable method to quantify prey habitat use. To achieve these goals, we used trace element analysis to quantify recruitment sources supporting predatory largemouth bass ( *Micropterus salmoides* ) and bowfin ( *Amia calva* ) directly (i.e., the predators) and indirectly (i.e., their consumed prey) in the Upper Mississippi River (UMR; Pools 4, 8, and 13). A previous study identified recruitment sources for common small‐bodied fish in this system (Valentine, Bouska, et al. 2024), which these predators happen to consume in high abundance (Valentine and Whitledge 2024). Whether the recruitment sources of predators are similar to the recruitment sources of prey, whether similarities in recruitment sources of consumed and independently sampled prey exist and whether recruitment sources differ across time and space were unknown. Specific objectives were to (1) identify the recruitment sources of individual predators and prey within each study reach (i.e., Pools 4, 8, and 13); (2) within a study reach, compare percentages of predators and prey recruited from each source; (3), across study reaches, compare percentages of predators or prey recruited from each source; (4) compare percentages of recruitment sources of consumed prey across years; and (5) compare percentages of recruitment sources of consumed prey to independently collected prey fish from Valentine, Bouska, et al. (2024), thereby testing the validity of using stomach contents as a way to determine prey recruitment sources.

## 2 Methods

### 2.1 Study Area and Sample Collection

**FIGURE 1:**
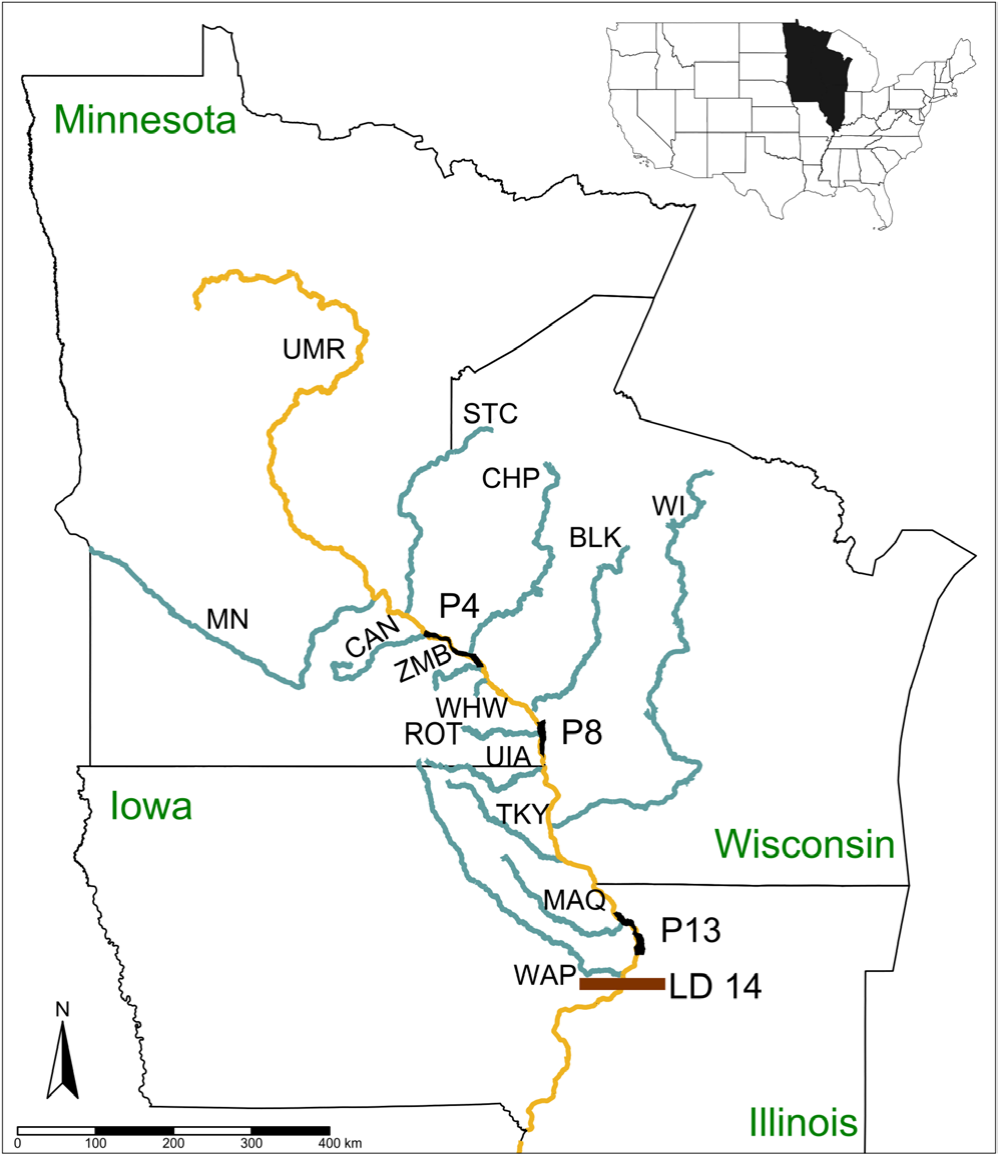
Map of the study area. The three study reaches are highlighted in black: Pool 4 (P4), Pool 8 (P8), and Pool 13 (P13). State names are given in green text. Lock and Dam 14 (LD 14) as the lower boundary of the system is shown with a brown box. The Upper Mississippi River (UMR) is shown in yellow and its tributaries are shown in blue. Tributary abbreviations: Minnesota (MN), St. Croix (STC), Vermillion (VER), Cannon (CAN), Chippewa (CHP), Zumbro (ZMB), Whitewater (WHW), Black (BLK), Root (ROT), Upper Iowa (UIA), Wisconsin (WI), Turkey (TKY), Maquoketa (MAQ), and Wapsipinicon (WAP) Rivers.

The UMR is the impounded portion of the Mississippi River, where 29 Lock and Dam structures have impounded this river into a series of navigation pools with altered flow and inundation patterns. This study encompassed three navigation pools of the UMR: Pool 4 near Lake City, Minnesota (river kilometer (RKM) 1210–1283), Pool 8 near La Crosse, Wisconsin (RKM 1092–1131), and Pool 13 near Bellevue, Iowa (RKM 841–896; Figure 1). Pools 4, 8, and 13 have complex habitats, including backwater habitats, submersed aquatic vegetation, lotic habitats, and slower‐moving waters (Koel 2001; Houser 2022) and generally have similar physical characteristics to one another (Koel 2001; Houser et al. 2010). Within these upper pools, many small and several large tributaries (i.e., Minnesota, Wisconsin, and St. Croix Rivers; discharge > 166 m^3^/s; Pracheil et al. 2013) influence physical characteristics. Within the UMR, we considered the mainstem river tributaries upstream from Lock and Dam 14 (RKM 800) as potential recruitment sources (Figure 1). In the UMR, open‐river conditions (i.e., dam gates are lifted out of the water) occur on average 9.2% (standard deviation (SD) 10.6%) of the year (Bouska 2021), creating potential opportunities for dispersal among pools beyond passage through the lock chambers (Fritts et al. 2021). However, both Lock and Dam 14 (1% of year) and 15 (5.4% of year) experience less frequent open‐river conditions (Bouska 2021), potentially constraining fish movement. We assumed a low probability of upstream movement from tributaries entering the UMR in Pool 14 and downstream (Valentine, Bouska, et al. 2024).

Personnel through the Long‐Term Resource Monitoring element of the Upper Mississippi River Restoration Program (LTRM) collected largemouth bass and bowfin from Pools 4, 8, and 13 from June 15 to October 31 during 2019 and 2020. Personnel used low‐pulse DC electrofishing as well as fyke nets (bowfin only) to collect largemouth bass and bowfin using standard LTRM sampling protocols described by Ratcliff et al. (2014). We collected additional bowfin, sciaenids (i.e., freshwater drum ( *Aplodinotus grunniens* )), and ictalurids from closed bodies of water and relied on data from previous studies (Smith and Whitledge 2011; Zeigler and Whitledge 2010; Laughlin et al. 2016) to characterize relationships between water and otolith chemistry. We assumed that fish from closed water bodies lived their entire lives in the collection site, so it was near certain that otolith chemistry reflected water chemistry. We removed, dried, and stored otoliths in microcentrifuge tubes for future analysis. Prey fish were identified in a diet analysis (Valentine and Whitledge 2024). We removed prey fish otoliths and cleaned them with nanopure water to remove digestive fluid; then, we dried and stored the otoliths in microcentrifuge tubes. We only analyzed prey fish from families that had a greater than 1% numerical abundance in the stomach contents among fish diet items (i.e., families Leuciscidae, Clupeidae, Centrarchidae, Percidae, Ictaluridae, and Sciaenidae; Valentine and Whitledge 2024).

### 2.2 Otolith Trace Element Analysis

Following protocols outlined by Valentine, Bouska, et al. (2024), we sectioned, sanded, polished, and mounted otoliths for trace element analysis. First, we embedded otoliths in Epofix epoxy resin. Second, we sectioned the embedded otoliths in the transverse plane using a Buehler IsoMet low‐speed saw to obtain a thin section containing the core. Third, to expose the otolith primordium and annuli, we sanded each thin section using 800 and 1000 grit sandpaper and polished it using lapping film. Finally, we mounted thin sections onto acid‐washed glass slides and stored slides in acid‐washed polypropylene Petri dishes (Laughlin et al. 2016; Valentine, Bouska, et al. 2024). We targeted at least 50 otoliths for analysis from each predator species (i.e., largemouth bass and bowfin) and pool (i.e., Pools 4, 8, and 13). For prey fish, we prepared one otolith from each identifiable fish prey using the same method as the predator otoliths.

We analyzed sectioned fish otoliths using laser ablation inductively coupled plasma mass spectrometry (LA‐ICPMS; Pracheil et al. 2014). Hard parts were analyzed for strontium (^88^Sr for Bowfin, ^86^Sr for all other species), magnesium (^25^Mg), and calcium (^43^Ca) using a Thermo X‐Series 2 ICPMS coupled with a CETAC technologies (Teledyne CETAC Technologies, Omaha, Nebraska) LSX‐266 LA system. We ablated a transect starting at 100 μm from the core of each otolith, passing through the core and extending to the opposite edge of the otolith (Laughlin et al. 2016; Whitledge et al. 2019; Valentine, Bouska, et al. 2024). This transect corresponded to the ontogenetic record of each fish's environmental history where the core represented early life environmental history (i.e., recruitment source), and the edge represented the most recent environmental history (i.e., capture location). The laser transect had a beam diameter of 25 μm, a scan rate of 5 μm/s, a pulse rate of 10 Hz, and an energy level of 35%. We preceded and succeeded each transect with a 30‐s Argon gas blank and a 30‐s washout period. After every 10–20 samples, we ablated three replicate 300‐μm transects of CaCO_3_ standard (United States Geological Survey, MACS‐3) to enable calculation of elemental concentrations from raw isotopic count data and to adjust for possible instrument drift (Laughlin et al. 2016; Valentine, Bouska, et al. 2024).

We converted elemental counts resulting from LA‐ICPMS to Sr:Ca. After correcting for instrument drift, the MACS‐3 matrix, and the argon gas blank, we converted Sr counts to concentrations (μg/g). We converted the resulting Sr concentrations (μg/g) to Sr:Ca (mmol:mol) by normalizing to Ca concentration based on Ca as the pseudo‐internal standard element (Laughlin et al. 2016; Whitledge et al. 2019; Valentine, Bouska, et al. 2024). We used the ElementR Shiny application (Sirot et al. 2016) for elemental conversions. All elemental concentrations were above the limits of detection.

### 2.3 Data Analyses

Prior to model creation and subsequent individual classification, we removed data from vateritic otoliths from analysis (Valentine, Bouska, et al. 2024). We determined vateritic otolith cores to have low Sr:Ca (e.g., < 150 μmol:mol) and high Mg:Ca (e.g., > 1000 μmol:mol) in combination (Tzeng et al. 2007). Strontium substitution rates in vaterite differ from aragonite, so our water–otolith chemistry relationships would not accurately classify fish environmental history from vateritic otoliths (Tzeng et al. 2007; Pracheil et al. 2019). Ten bowfin and one leucisid prey had vateritic otoliths and were removed from analysis.

#### 2.3.1 Water‐Otolith Chemistry Relationships

**FIGURE 2:**
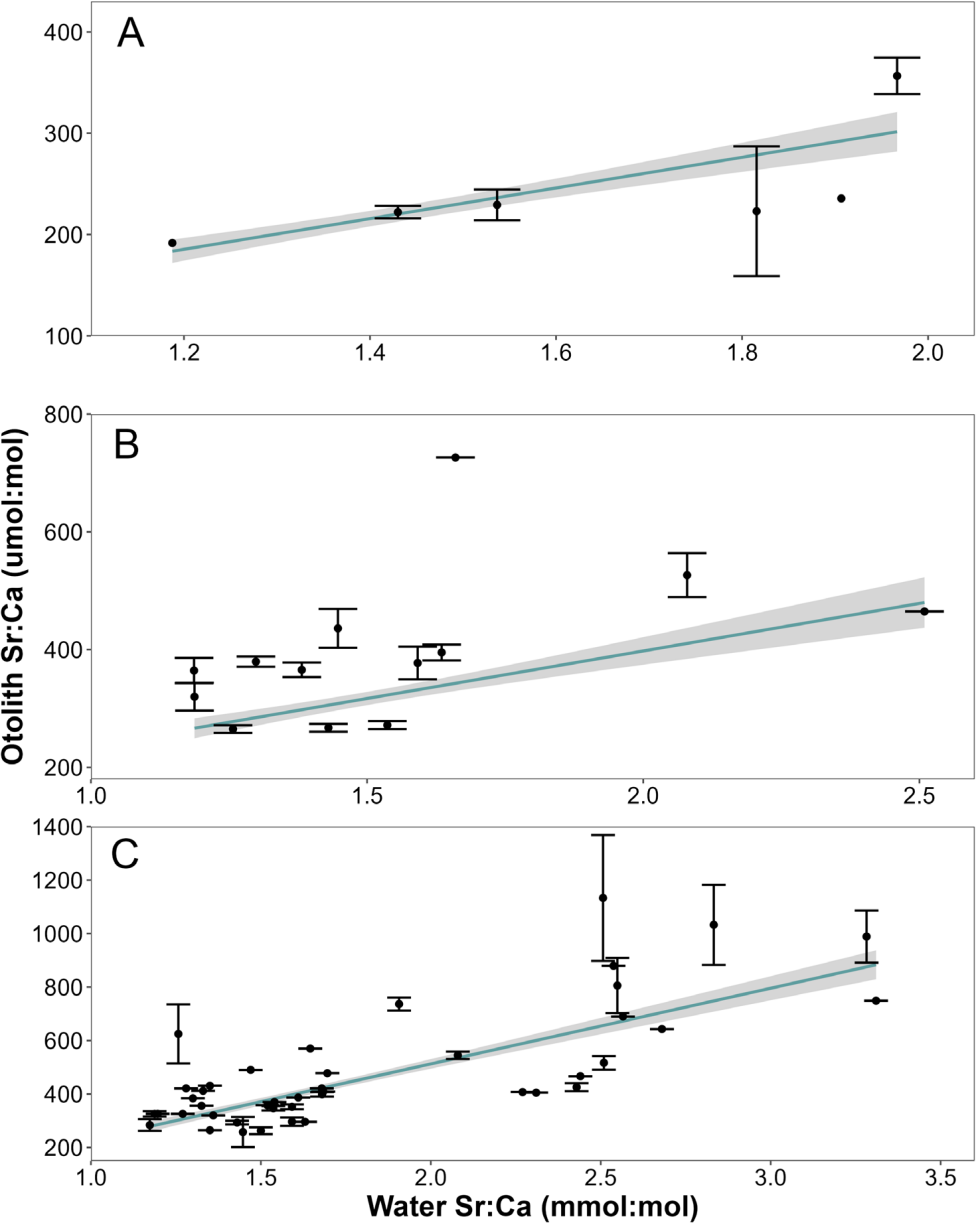
Variability in otolith edge Sr:Ca and average water Sr:Ca for bowfin (A), sciaenids (B), and ictalurids (C). Data points are mean otolith edge chemistries for each water body sampled and error bars represent ± standard error. Blue lines act as visuals of the relationship between otolith Sr:Ca and water Sr:Ca using a simple linear model function.

We used previously published or developed family‐specific water‐otolith chemistry relationships to classify fishes to potential recruitment sources. Previously published otolith‐water Sr:Ca relationships predicted expected ranges of otolith chemistry for each potential water body (i.e., mainstem river or tributary) in the study area for centrarchids, percids, clupeids, and leuciscids (Valentine, Bouska, et al. 2024). For all families, we assumed a linear relationship between otolith and water Sr:Ca, as other studies have shown linear relationships for other species (Walther and Thorrold 2008; Radigan et al. 2018; Whitledge et al. 2021; Valentine, Whitledge, et al. 2024). Mean Sr:Ca of the last 50 μm of the ablated otolith transect corresponded to the edge chemistry of each otolith, and we assumed that this otolith chemistry corresponded to the water chemistry of the environment of capture. Ictalurids, sciaenids, and bowfin did not have published water‐otolith chemistry relationships that controlled for variability among sampling locations. We developed a relationship for ictalurids and sciaenids using mixed effects linear models between water chemistry and otolith chemistry of collected individuals from the UMR and closed water bodies using archived water chemistry data (Valentine, Whitledge, et al. 2024). We used sampling location as a random effect and average water chemistry as a fixed effect to predict otolith edge chemistry (Valentine, Bouska, et al. 2024). Additionally, we included sampling location as a weight to reduce the influence of outliers in model creation, which led to reduced Akaike's information criterion for models. For bowfin, we used partition coefficients to determine the relationship between water and otolith Sr:Ca. Bowfin were sampled from water bodies with a narrow Sr:Ca range, leading to poor predictability for bowfin origins or inappropriate classification methods if we extrapolated resulting models. We followed methods in Whitledge et al. (2021) to create partition coefficients between bowfin otolith Sr:Ca and water body Sr:Ca for each water body of capture. The partition coefficient was the ratio of the mean otolith edge Sr:Ca (μmol:mol) for individuals within the water body to the mean water Sr:Ca (mmol:mol) for the water body (Figure 2A).

We used the resulting or published models and partition coefficients to predict the expected otolith chemistry ranges for each tributary and the UMR. To avoid issues with extrapolating predicted otolith chemistry values from models (Hanh 1977), we calculated upper and lower limits of otolith Sr:Ca by location only when we could do so within the bounds of the taxa‐specific water‐otolith chemistry models. Using archived water chemistry data (Valentine, Bouska, et al. 2024), we calculated predicted otolith chemistry ranges from the 5th and 95th percentiles of Sr:Ca water chemistry profiles (Laughlin et al. 2016; Rude and Whitledge 2019; Whitledge et al. 2019; Valentine, Bouska, et al. 2024) using the model equations. For bowfin, we multiplied the lowest (96.9 otolith: water Sr:Ca) and highest (178 otolith: water Sr:Ca) partition coefficient values by the 5th and 95th percentiles of Sr:Ca water chemistry profiles, respectively, for each water body (Whitledge et al. 2021). These ranges corresponded to expected otolith chemistries for each river where the minimum and maximum values represented thresholds of minimum and maximum otolith chemistry values.

#### 2.3.2 Natal Origin Classification

We classified each predator and prey fish to potential recruitment sources. To determine recruitment source, we compared the average Sr:Ca of first 50 μm centered on the otolith core to the predicted otolith Sr:Ca for each river similar to methods outlined in Valentine, Bouska, et al. (2024). For fishes that had otolith core chemistry outside of the predicted mixed effects model limits for otolith chemistry, we determined these fishes to be “unclassifiable” and noted whether individuals had a mean otolith core Sr:Ca above or below the model limits. Due to overlap in water Sr:Ca, some fishes classified into multiple tributaries or the mainstem river. Therefore, we classified individual fishes as “tributary” or “potential resident” origin. Tributary origin individuals only classified to tributaries, whereas potential resident origin individuals classified into at least one tributary and the UMR. Because some tributaries had distinct water chemistry, we refined these general category classifications when possible to specific rivers (Valentine, Bouska, et al. 2024). However, due to uncertainty in origin, for tributary origin individuals, those that classified into three or more tributaries and whose confluences with the UMR span multiple reaches were grouped into a “tributary” category. To reduce the number of categories for easier comparison, individuals that classified to the Wisconsin River and one or two other tributaries throughout the UMR were classified as “small tributary or Wisconsin River.” For tributary origin individuals, if a mean otolith core Sr:Ca fell outside of the range of all known water bodies' predicted otolith chemistry and was within the model limits, we noted whether this otolith had low or high Sr:Ca. For potential residents that classified into three or more tributaries and the UMR, we classified them as “tributary or UMR” origin (Valentine, Bouska, et al. 2024).

#### 2.3.3 Origin Comparison

We determined if natal origin classifications differed across species, study reaches, and collection years using Fisher exact tests (Whitledge et al. 2019). For these analyses, we used the refined natal origin categories (e.g., specific tributaries). First, we tested if the percentages of predators classified to each natal origin category differed between largemouth bass and bowfin and among study reaches within a species. Second, we repeated these classification comparisons across consumed prey families and within a prey family among study reaches. For this analysis, we removed sciaenids because sample sizes were too low for comparison (i.e., < 5 individuals per study reach). Third, we tested if percentages of consumed prey classified to each natal origin category varied between sampling years within each study reach. Specifically, we grouped all consumed prey fish by year and study reach to compare those collected in 2019 with those collected in 2020.

Finally, we used Fisher exact tests to test whether consumed and independently sampled prey fish from Valentine, Bouska, et al. (2024) differed in origin classification. To compare the same natal origin categories across studies, we reclassified the Upper Iowa or Wisconsin category in Valentine, Bouska, et al. (2024) to “small tributary or Wisconsin River” and removed fish from the comparison that had a mean otolith core Sr:Ca above or below the model limits. We compared taxa in this study – leuciscids, centrarchids, percids, and clupeids – to emerald shiner ( *Notropis atherinoides* ) and bullhead minnow ( *Pimephales vigilax* ; leuciscids), bluegill ( *Lepomis macrochirus* ; centrarchids), yellow perch, (*Perca flavescens*; percids), and gizzard shad (*Dorosoma cepedianum*; clupeids) origins from Valentine, Bouska, et al. (2024). We used a Bonferroni correction for multiple comparisons (0.05/number of tests) and conducted analyses in R version 4.1.2 (R Core Team 2023).

## 3 Results

### 3.1 Water‐Otolith Chemistry Relationships

We did not model the relation between water and otolith Sr:Ca for Bowfin, but it appeared to be linear (Figure 2A), following our partition coefficient assumptions. Sciaenid and ictalurid otolith Sr:Ca were positively related to water Sr:Ca (Sciaenidae: *y* = 337.04(*x*) − 131.55, *F*_1,18_ = 242.20, marginal coefficient of determination (*R* ^2^) = 0.607, conditional *R* ^2^ = 0.696, *p* < 0.001, Figure 2B; Ictaluridae: *y* = 196.04(*x*) + 71.30, *F*_1,47_ = 330.25, marginal *R* ^2^ = 0.522, conditional *R* ^2^ = 0.650, *p* < 0.001, Figure 2C).

### 3.2 Natal Origin Classification

**FIGURE 3:**
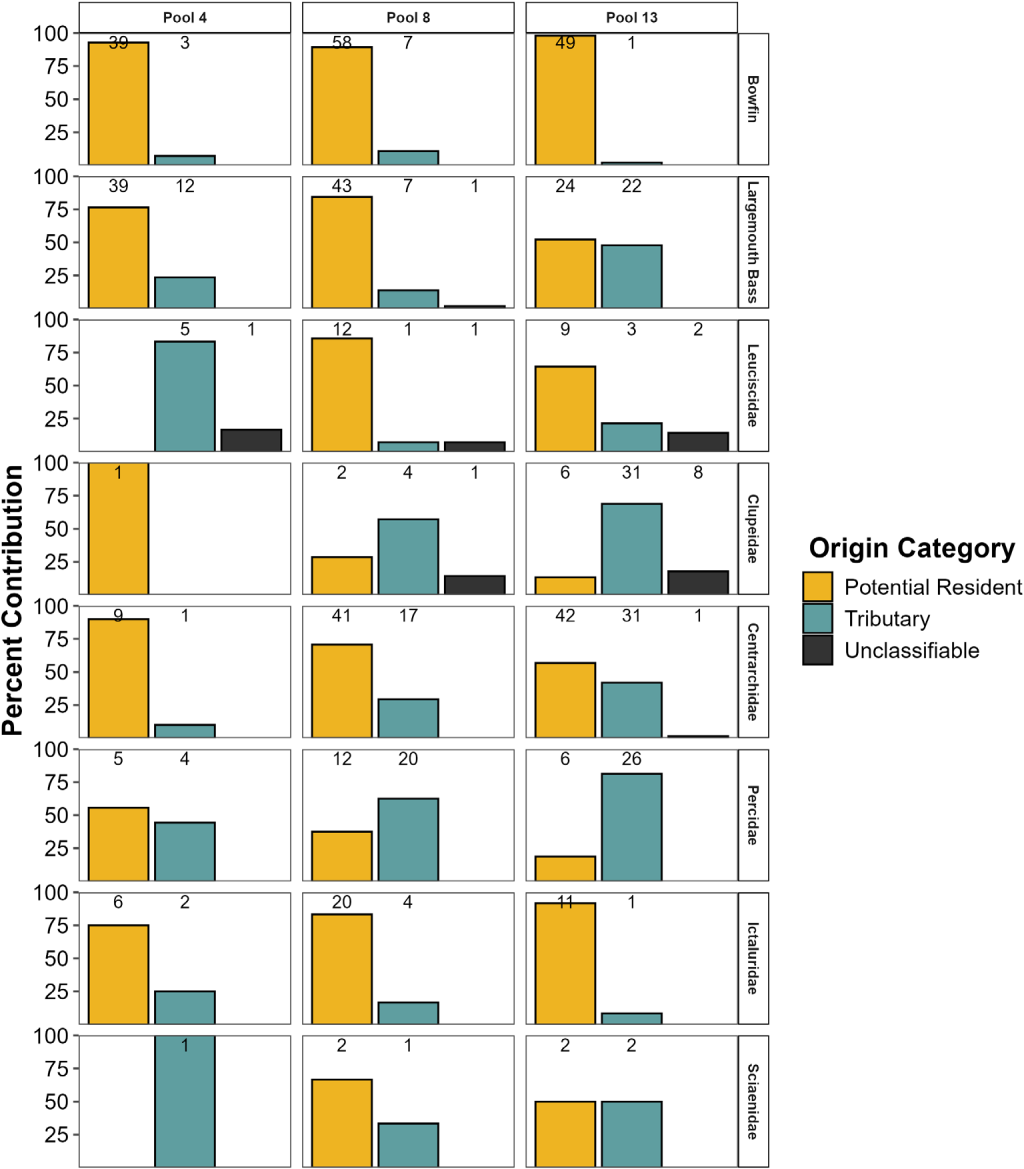
Predator and prey natal origin classification to origin categories (potential resident and tributary) across study reaches. Numbers above columns represent the number of individuals classified to each category.

For predators, most largemouth bass and bowfin classified as potential residents (Figure 3). Among all predators combined, 83% classified as potential residents and 17% as tributary origin. One largemouth bass in Pool 8 had an otolith chemistry outside of the model limits and was therefore unclassifiable. Among all study reaches, most largemouth bass and bowfin classified as potential residents: in Pool 4, 93% of bowfin and 76% of largemouth bass; in Pool 8, 89% of bowfin and 84% of largemouth bass; and in Pool 13, 98% of bowfin and 52% of largemouth bass. Among all study reaches, only 2%–11% of bowfin but more (14%–48%) largemouth bass classified as tributary origin.

Across study reaches, 35 prey from Pool 4, 138 prey from Pool 8, and 182 prey from Pool 13 classified to natal origin (Figure 3). Among all prey combined, 53% classified as potential residents, 44% as tributary origin, and 4% as unclassifiable. Only leuciscid, clupeid, and centrarchid prey were unclassifiable in relatively low numbers across study reaches (Figure 3). In Pool 4, all sciaenids and leuciscids and 10%–44% of individuals in other families classified to tributary origin. In Pool 8, 57% of clupeids, 63% of percids, and 7%–33% of individuals in other families classified to tributaries. Pool 13 had a similar pattern to Pool 8: 69% of clupeids, 81% of percids, and 8%–50% of individuals from other families classified to tributary origin (Figure 3).

**FIGURE 4:**
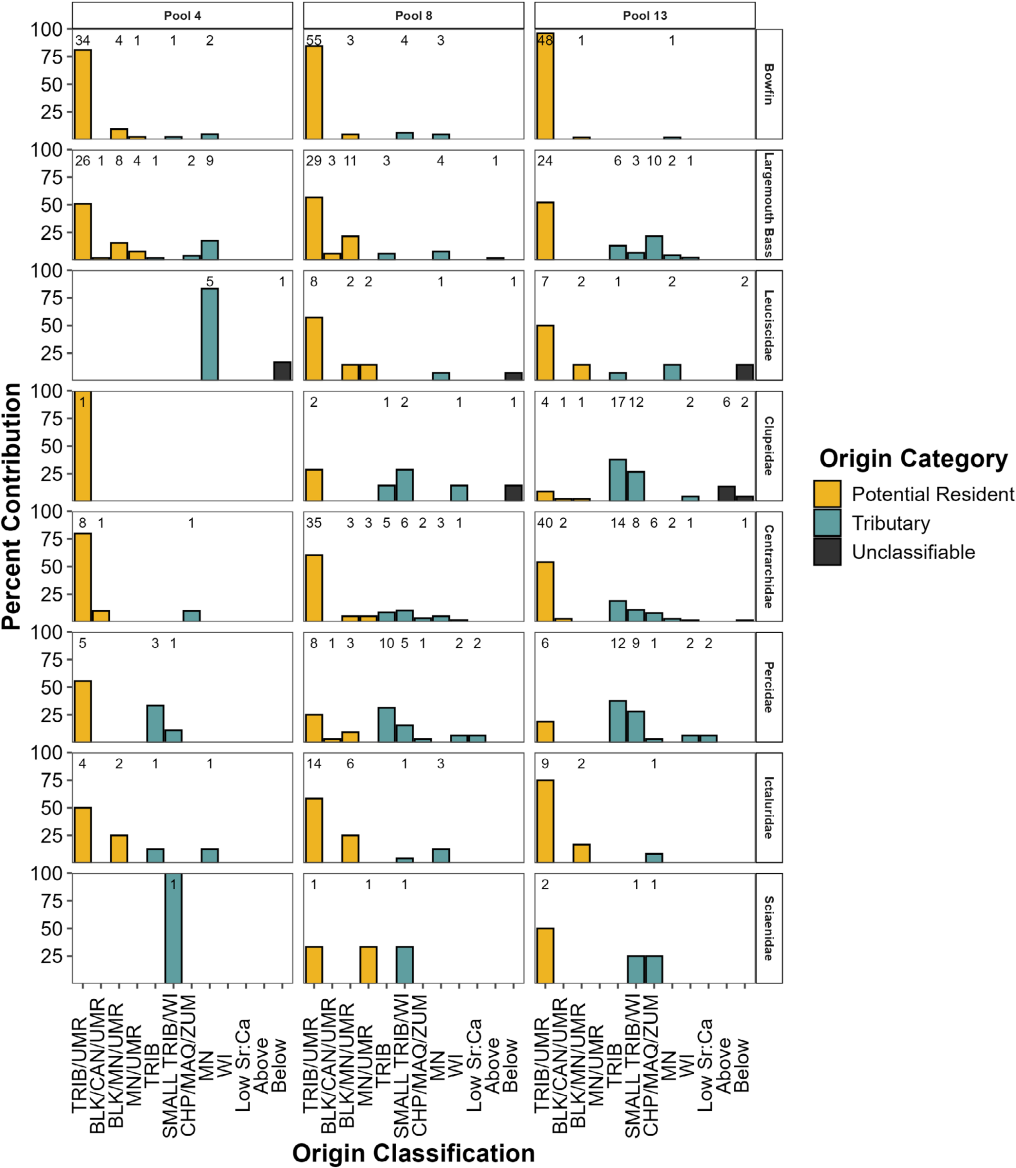
Natal origin classification of predators and consumed prey collected in the three study reaches (Pools 4, 8, and 13). Numbers above columns represent number of individuals classified to each category. Upper Mississippi River (UMR). Tributary (TRIB) abbreviations: Minnesota (MN), Cannon (CAN), Chippewa (CHP), Zumbro (ZMB), Whitewater (WHW), Black (BLK), Wisconsin (WI), and Maquoketa (MAQ) Rivers. Above and below refer to individuals with a core otolith chemistry above or below model limits. Low Sr:Ca refers to individuals with a low Sr:Ca otolith core chemistry within model limits but that did not classify to a river in this study region.

Fish classified to multiple refined natal origin classification categories (Figure 4) indicated that multiple habitats (e.g., tributaries and mainstem river) contributed to the recruitment of predators and prey. Both large (e.g., Minnesota and Wisconsin Rivers) and small (e.g., Chippewa, Maquoketa, and Zumbro Rivers) tributaries contributed recruits of largemouth bass and prey species across all study reaches. Details of refined natal origin classifications that are study reach specific for both predators and prey are in Appendix A.

### 3.3 Origin Comparisons

**FIGURE 5:**
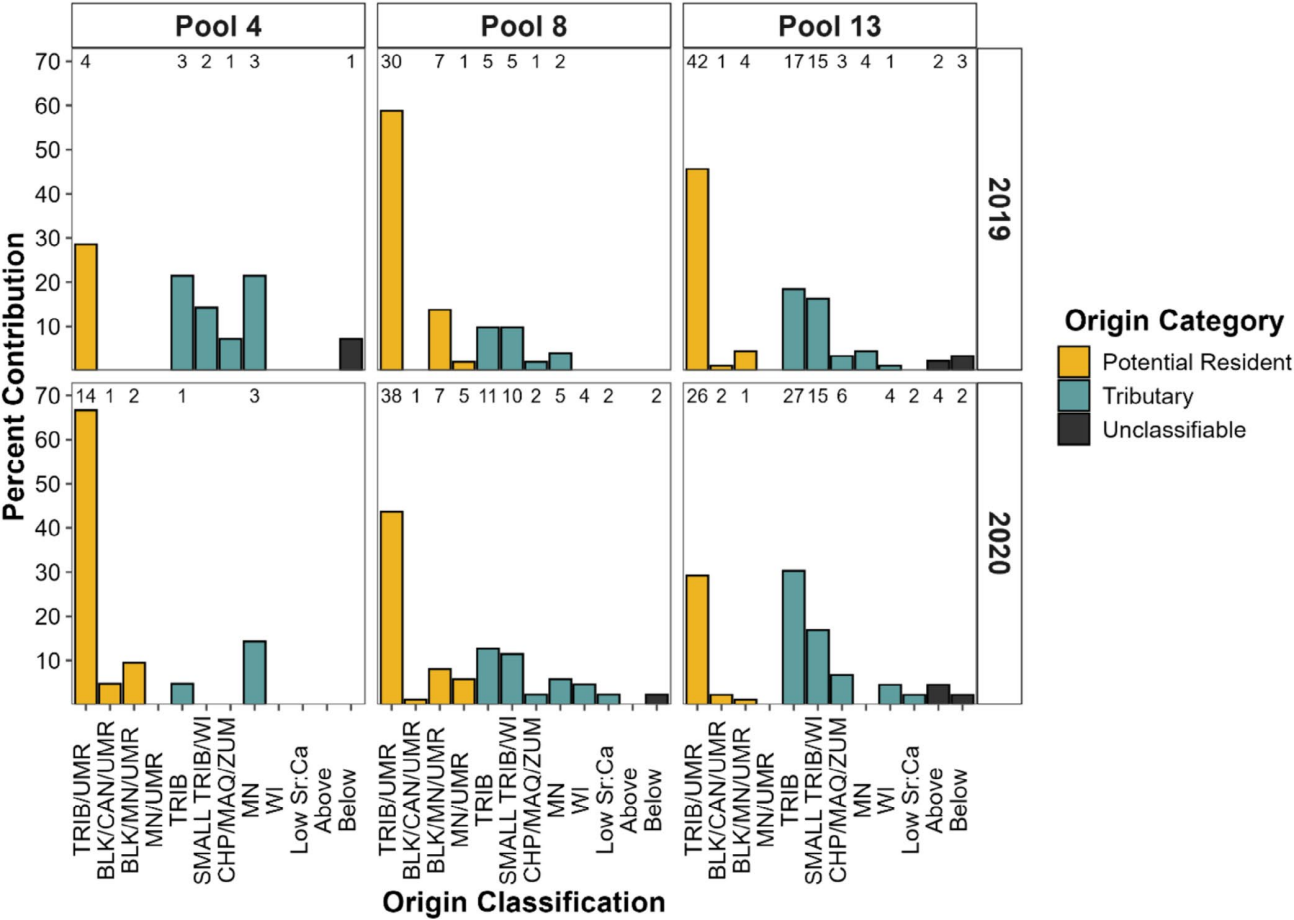
Natal origin classification of prey fishes combined across families collected in the stomachs of predators from the three study reaches (Pools 4, 8, and 13) by year of predator collection. Numbers above columns represent number of individuals classified to each category. Upper Mississippi River (UMR). Tributary (TRIB) abbreviations: Minnesota (MN), Cannon (CAN), Chippewa (CHP), Zumbro (ZMB), Whitewater (WHW), Black (BLK), Wisconsin (WI), and Maquoketa (MAQ) Rivers. Above and below refer to individuals with a core otolith chemistry above or below model limits. Low Sr:Ca refers to individuals with a low Sr:Ca otolith core chemistry within model limits but that did not classify to a river in this study region.

**FIGURE 6:**
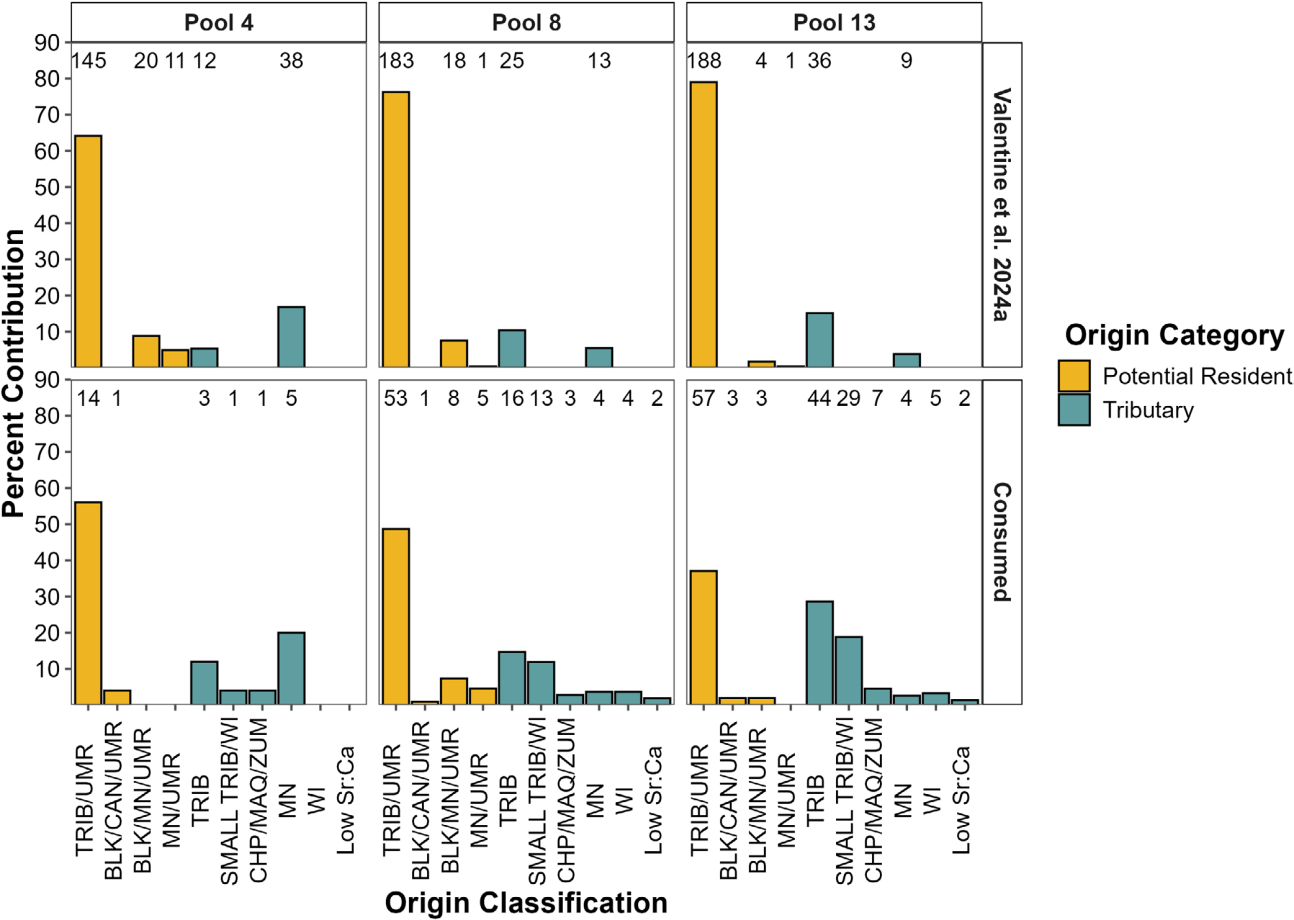
Natal origin classification of prey fishes combined across families collected in the stomachs of predators and from the wild previously analyzed by Valentine, Bouska, et al. (2024) from the three study reaches (Pools 4, 8, and 13). The numbers above columns represent the number of individuals classified to each category. Upper Mississippi River (UMR). Tributary (TRIB) abbreviations: Minnesota (MN), Cannon (CAN), Chippewa (CHP), Zumbro (ZMB), Whitewater (WHW), Black (BLK), Wisconsin (WI), and Maquoketa (MAQ) Rivers. Low Sr:Ca refers to individuals with a low Sr:Ca otolith core chemistry within model limits but that did not classify to a river in this study region.

All pairwise comparisons among study reaches and species were statistically similar (*p*‐values > 0.71). These similarities indicated that origin classifications were similar between predator species, among consumed prey families, across study reaches for predators and consumed prey, between sampling years (Figure 5), and between consumed and independently sampled prey (Figure 6).

## 4 Discussion

We found that mainstem river predator and prey fish assemblages recruited from a variety of habitats and in particular tributaries, indicating that connectivity exists among the mainstem river and its tributaries. This finding highlighted that tributaries may disproportionately support predator production through directly contributing recruits and indirectly contributing their prey past early life stages. Tributary contributions to predator and prey fish recruits were consistent across space, time, and trophic position. Spatiotemporally stable patterns indicate that connectivity among tributary and mainstem river habitats may be important for the long‐term persistence of multi‐trophic level fish assemblages in the UMR. Further, predators and their prey recruited from different sources as tributary identities differed somewhat, indicating that a large portfolio of tributaries contributes to predator and prey assemblages. Combined, we extended the growing body of literature on environmental use and history of fishes within large river systems (Whitledge et al. 2007; Pracheil et al. 2014, 2019; Norman and Whitledge 2015; Laughlin et al. 2016; Radigan et al. 2018; Valentine, Bouska, et al. 2024) with an emphasis on environments that support predators. We also noted that using stomach contents of predators was a viable technique for classifying the environmental history of fishes across multiple trophic levels using only predator sampling.

We identified some tributaries that consistently lent recruits, supporting both predators and small‐bodied fish assemblages in the UMR. Across reaches, fish classified to the Minnesota River, similar to results from Valentine, Bouska, et al. (2024). In the current study, we also found relatively high percentages of tributary origin individuals that classified to small tributaries or the Wisconsin River. High classification to these refined origin categories may be a consequence of their unique high (i.e., Minnesota River) or low (e.g., Wisconsin, and Zumbro Rivers) Sr:Ca (Valentine, Whitledge, et al. 2024). These rivers may also have contributed more recruits than classified: unclassifiable individuals with otolith chemistry above and below the model limits likely originated from the Minnesota River or low Sr:Ca rivers, respectively. These rivers may provide crucial spawning and early life rearing conditions for prey fishes that are unique in the UMR system like the combination of riffle, low gradient, and floodplain habitats in the Minnesota River (Kirsch et al. 1985), which has led to abundance of prey fishes including gizzard shad, bluegill, black bullhead ( *Ameiurus melas* ), and emerald shiner in the downstream floodplain reaches (Schmidt and Polomis 2007). These origins also indicated that the mainstem river was connected to downstream (Minnesota River) and perhaps upstream (Wisconsin River) tributaries. Minnesota River recruits sampled in Pool 13 moved downstream more than 500 RKM. Such long‐distance, pervasive effects of single rivers in a network are not unheard of in this (Laughlin et al. 2016; Valentine, Bouska, et al. 2024) and other rivers (Stuart and Sharpe 2020; Bouska et al. 2023). These results support the use of interjurisdictional management and a lens of macro‐structuring of the UMR and its tributaries. To quantify the prevalence of these patterns among the world's rivers, more research could quantify large‐scale movements (Stuart and Sharpe 2020) and use of multiple habitats across the lives of fish or other mobile species (Duncan et al. 2021; Vivancos et al. 2021).

The mainstem river may provide critical habitat for recruitment of UMR fish assemblages. Most fishes classified as potential residents, which indicated two potential scenarios. First, tributaries may lend fewer recruits to the UMR predator and prey assemblage than mainstem habitats. This theory aligns with known breeding and early life habitat use of bowfin and largemouth bass. Both species similarly use vegetated backwater or low‐velocity habitats for breeding and early‐life parental care (Pflieger 1997). Greater availability and accessibility of vegetated habitats may occur in large rivers like the UMR and the Minnesota River than in smaller rivers. Indeed, our study reaches contain vegetated backwaters and side channels (Houser 2022) and have experienced increased submersed aquatic vegetated habitats recently (De Jager and Rohweder 2017). Alternatively, tributaries may lend more recruits than we discerned. Overlapping Sr:Ca among the UMR and many tributaries led to high classification percentages of potential residents (i.e., classified to mainstem river and tributaries). Likely, at least some potential residents originated from tributaries, especially where wetland complexes with aquatic vegetation exist, such as tributary confluences (Kirsch et al. 1985). Therefore, tributary origin percentages should be considered minimum percentages.

Multiple habitat types and connectivity among them contributed to the production of predators both directly and indirectly. Directly, some predators classified as known tributary origin, especially largemouth bass in Pool 13. Indirectly, over a quarter of all prey classified as known tributary origin. Even though the direct contributions of tributaries were lower for predators, bowfin and largemouth bass rely on prey fish as food subsidies, and due to their high trophic positions, they also experience cumulative effects of alterations in prey assemblages (Vázquez and Simberloff 2002). Thus, because tributaries contributed to their prey sources, predators may rely more heavily on connectivity among tributaries and the mainstem river than their own recruitment sources indicated. Additionally, across multiple trophic levels, similar tributaries (e.g., Minnesota and Wisconsin Rivers) contributed recruits, and the sources of recruits were relatively diverse as fish originated from multiple potential resident and tributary categories. These results indicate that the recruitment of predators and prey was coupled with one another temporally and across heterogeneous space, which could lead to stability of the assemblage (White 2007; Townsend and Gouhier 2019; Pettersson and Jacobi 2021). Notably, this coupling occurred past the early life history stages (e.g., larvae) within this assemblage to fuel predator production across multiple size classes (Valentine and Whitledge 2024).

Origin patterns were consistent temporally and spatially for this UMR fish assemblage. Large‐scale similarities are consistent with species‐specific conclusions in this river (Laughlin et al. 2016; Pracheil et al. 2019; Valentine, Bouska, et al. 2024) and other systems (Collins et al. 2013; Spurgeon et al. 2018; Duncan et al. 2021; Vivancos et al. 2021). Temporally, our results indicated that at small time scales and among these species, consistent recruitment sources contributed to prey assemblages in the mainstem UMR. Samples were collected in hydrologically different years: 2019 had persistent high discharge conditions throughout the growing season, whereas 2020 had a more typical hydrograph for the upper pools (Van Appledorn 2022), indicating that tributary contributions may remain consistent even in the face of stochastic events. Although we could not consistently determine the identity of the specific tributaries contributing recruits to know if the same tributaries lend recruits across years and reaches, it appears that at least some rivers (e.g., Minnesota River) consistently lent recruits. Other tributaries may also consistently lend recruits, or recruitment sources could vary spatiotemporally across the portfolio of tributaries to yield consistent contributions to the mainstem river. Spatially, prey and predators had statistically similar origins among study reaches, indicating that connectivity among the mainstem UMR and tributaries allows for dispersal of organisms, despite the presence of modifications (e.g., Lock and Dam structures). Spatial similarities corroborate previous genetics work that indicates some of these UMR fishes that have panmictic populations (Shi et al. 2023). Combined, consistency among origin patterns indicates that at a large scale, tributaries and their connectivity with the mainstem river were important for the entire predator–prey assemblage of the UMR. Further comparison across longer time spans could elucidate long‐term patterns that drive ecological structuring across trophic levels, persistence of assemblages in the face of environmental stochasticity and perturbations, and the degree portfolio effects lead to long‐term coupling of predators and prey (Collins et al. 2013; Brennan and Schindler 2017; Harrison et al. 2020).

Species‐specific habitat associations and life history strategies may affect specific origin patterns. Although prey and predators had statistically similar origin categories, the identity of origin classifications differed somewhat among species. Percids and centrarchids, which had relatively high percentages of tributary origin individuals, use vegetation and low velocity water bodies for spawning and early life history (Pflieger 1997). These types of habitats exist in the UMR (Houser 2022), providing opportunities for spawning and nursery grounds. These habitats likely also exist in tributaries and near the confluences of tributaries as Valentine, Bouska, et al. (2024) suggest, and confluences likely have tributary chemical signatures. Fishes may flush from these confluences and enter the UMR as described by previous direct observations (Holland and Sylvester 1983). Clupeids (gizzard shad) classified most often to tributaries potentially because they use floodplain habitats and backwaters of tributaries where they congregate as young (Holland and Sylvester 1983), similar to percids and centrarchids. Other prey such as *Etheostoma* darters (Percidae) require a cobble substrate (Pflieger 1997) typically found in smaller streams. Small tributaries likely contributed recruits of these percids to the mainstem river (e.g., general tributary category), and habitat connectivity likely would be important to these fish long‐term (Duncan et al. 2021; Vivancos et al. 2021). However, the identity of potential small tributaries that recruited these fishes to the mainstem river could not be identified using otolith Sr:Ca alone. Despite low sample sizes, sciaenid (freshwater drum) origin patterns were mixed, which may stem from their migratory and broadcast spawning traits (O'Hara et al. 2007). Leuciscids (shiners and minnows) tend to be large‐river specialists (O'Hara et al. 2007), and their origin patterns reflected this affinity as almost all tributary origin individuals classified to the large Minnesota River. Finally, past research indicates that ictalurids originated from mainstem habitats within the Middle Mississippi River (Laughlin et al. 2016). Our results align with Laughlin et al.'s (2016) to further highlight the potential use of mainstem habitats as recruitment sources to ictalurids through the high percentages of potential resident ictalurids. Perhaps, the mainstem river in the UMR provides sufficient habitat for ictalurid recruitment.

Using stomach contents, we effectively evaluated patterns in origins of prey and predators simultaneously. Origin patterns were statistically similar between independently sampled prey fishes from the UMR (Valentine, Bouska, et al. 2024) and consumed prey fishes. Our findings extend the applications of studying predators and prey simultaneously in previous marine (Lenanton et al. 2003; Kemp et al. 2011) and laboratory studies (Phelps et al. 2009). This comparison indicates several important points regarding predator–prey dynamics in the UMR. First, bowfin and largemouth bass likely consumed prey in proportion to what they encountered in the environment, corroborating their known opportunistic feeding habits (Valentine and Whitledge 2024). Second, prey from multiple origins likely mix in the mainstem UMR to occupy the same habitats rather than form clusters of individuals with similar origins, which likely reflects broadcast spawning habits (O'Hara et al. 2007) and panmictic populations (Shi et al. 2023). Finally, using stomach contents of predators can reduce sampling effort and organism sacrifice while capturing ecological variation. Further use of this technique could quantify the ecology of multi‐trophic level assemblages by providing information on hard to sample or rare species consumed by predators.

Several factors limited the interpretation of our data. First, due to small sample size and inability to identify all prey to species, we grouped prey by family, which may have occluded unique patterns among species with differing life history characteristics (e.g., *Etheostoma* spp. and yellow perch in Percidae). Second, the paucity of bowfin from water bodies that differed in Sr:Ca hindered our ability to create a robust model for precise classification. The use of partition coefficients allowed us to classify bowfin, but this approach was quite conservative and lacked precision. Third, some fish had otolith chemistry within model bounds that did not correspond to a water body because the rivers in this study were not exhaustive of all UMR tributaries. More water chemistry data could be collected to capture variability in all potential recruitment sources to fill classification gaps. Fourth, varying degrees of overlap in Sr:Ca among water bodies prevented further refinement of natal origin classifications. Use of Ba:Ca as a second natural marker was not feasible for these fish due to high within‐group variability compared to between‐group variability (Zeigler and Whitledge 2010). Future studies could consider the potential utility of strontium isotopes (e.g., ^87^Sr/^88^Sr) as an additional marker to refine fish origin assignments in the UMR. Finally, early life movements away from an individual's hatching location could lead to a mismatch between true natal origin and observed natal origin. For example, if fishes hatched in a tributary and quickly drifted out of the tributary to the mainstem UMR, tributary chemical signatures would likely not impart in the otolith core (Campana and Thorrold 2001; Pracheil et al. 2014), leading to high mainstem river (i.e., potential resident) classification rates, underestimating the contribution of tributaries and connectivity among habitats.

## 5 Conclusions

We highlighted the direct and indirect importance of tributaries and connectivity among tributaries and the mainstem river to predators in a large river. Our findings underscored consistency in recruitment sources across multiple trophic levels, space, and time. Additionally, we showed the utility of using predator stomach contents to simultaneously describe ecological patterns of predators and prey. Our results, in conjunction with results from Valentine, Bouska, et al. (2024), indicate that maintaining habitat connectivity within the UMR may lead to the persistence of predator and prey fish assemblages, and tributaries (e.g., Minnesota River) may play a vital role in fish recruitment. Thus, interjurisdictional management and maintaining or enhancing the availability of crucial habitats for fishes to meet their life history requirements may be the next steps for multi‐trophic level management in this river. However, we do not know whether life‐long movements of fishes require continued habitat connectivity (Duncan et al. 2021), if origins shift across long periods of time, if origin patterns reflect all fishes, and if these results are translatable to other river systems. To address these knowledge gaps, future efforts could examine life‐long movements of predators and prey, use archived samples for long‐term observations, include longer‐lived and migratory species, and determine how small percentages of recruits from specific locations may lead to a portfolio effect and influence population dynamics of target assemblages.

## Author Contributions

**Shaley A. Valentine:** conceptualization (lead), data curation (lead), formal analysis (lead), investigation (lead), methodology (equal), project administration (equal), validation (equal), visualization (lead), writing – original draft (lead), writing – review and editing (lead). **Kristen L. Bouska:** conceptualization (equal), funding acquisition (lead), supervision (supporting), writing – review and editing (supporting). **Gregory W. Whitledge:** conceptualization (equal), funding acquisition (supporting), methodology (equal), project administration (equal), resources (lead), supervision (lead), writing – review and editing (supporting).

## Conflicts of Interest

The authors declare no conflicts of interest.

## Data Availability

Data are available at https://doi.org/10.5066/P1YFYSUD.

## Appendix A

### Refined Results

#### Refined Natal Origin Classification

##### Pool 4

Most predators classified as potential residents from general tributaries or the Upper Mississippi River (UMR; 8% bowfin ( *Amia calva* ), 51% largemouth bass ( *Micropterus salmoides* ); Figure 4). Only one largemouth bass (2%) classified to the Black, Cannon, or Upper Mississippi Rivers. Among other potential resident categories, 10% of bowfin and 16% of largemouth bass classified to the Black, Minnesota, or Upper Mississippi Rivers, and one (2%) bowfin and 8% of largemouth bass classified to the Minnesota River or the UMR. Among those with a tributary origin classification, only one largemouth bass (2%) classified to the general tributaries category. Among specific tributaries, predators most often classified to the Minnesota River (5%–18%). Predators classified less frequently to other tributary categories: one bowfin classified to small tributaries or the Wisconsin River, and two (4%) largemouth bass classified to the Chippewa, Maquoketa, or Zumbro Rivers.

Consumed prey classified most often to the general tributary or UMR category (Figure 4). Additionally, one centrarchid (10%) and two percids (25%) classified to the Black, Cannon, or Upper Mississippi Rivers and the Black, Minnesota, or Upper Mississippi Rivers categories. Tributary classifications were widespread among categories. One ictalurid (13%) and 33% of percids classified to general tributaries, one percid (11%) and one sciaenid (100%) classified to small tributaries or the Wisconsin River, and one centrarchid classified to the Chippewa, Maquoketa, or Zumbro category. All classifiable leuciscids and one ictalurid (13%) classified to the Minnesota River. Only one leuciscid was unclassifiable with a Strontium to Calcium ratio (Sr:Ca) below the model limits.

##### Pool 8

Most predators classified as potential residents from general tributaries or the UMR (57% largemouth bass, 85% bowfin; Figure 4). Only 6% of largemouth bass classified to the Black, Cannon, or Upper Mississippi Rivers, whereas 5% of bowfin and 22% of largemouth bass classified to either the Black, Minnesota, or Upper Mississippi Rivers. Among tributary origin individuals, 6% of largemouth bass classified to the general tributary category, 6% of bowfin classified to small tributaries or the Wisconsin River, and 5%–8% of each predator classified to the Minnesota River. The one unclassifiable largemouth bass (2%) had an otolith Sr:Ca above the model limit.

Consumed prey most often classified to the general tributary or UMR category (29%–60%; Figure 4). Only one percid classified to the Black, Cannon, or Minnesota Rivers category and up to a third of individuals classified to the Black, Minnesota, or UMR and Minnesota or UMR categories. Among tributary origin individuals, up to one‐third of individuals classified to general tributaries (percids) or to small tributaries or the Wisconsin River (sciaenid and clupeid), and 14% or less of individuals classified to other tributary categories. One leuciscid and one clupeid were both unclassifiable with an otolith Sr: Ca below the model limits.

##### Pool 13

Pool 13 had the highest percentage of tributary origin predators among pools. Most predators classified as potential residents in the general tributaries or UMR category (96% bowfin, 52% largemouth bass; Figure 4). One bowfin (2%) classified to the Black, Cannon, or Upper Mississippi Rivers category. Among largemouth bass tributary classifications, 13% classified to general tributaries, 9% to small tributaries or the Wisconsin River, 20% to the Chippewa, Maquoketa, or Zumbro Rivers, 4% to the Minnesota River, and one (2%) to the Wisconsin River. One (2%) bowfin classified to the Minnesota River.

Of potential resident consumed prey, 9%–75% classified to general tributaries or the UMR (Figure 4). Few individuals (< 7%) classified to the Black, Cannon, or Upper Mississippi Rivers and the Black, Minnesota, or Upper Mississippi Rivers categories. Among tributary origin individuals, upwards of 38% of individuals (e.g., clupeids and percids) classified to general tributaries and small tributaries or the Wisconsin River categories. Fewer (< 25%) classified to other tributary categories. Among unclassifiable individuals, six clupeids had an Sr:Ca above the model limits, and all others (two leuciscids, two clupeids, and one centrarchid) had otolith Sr:Ca below the model limits.
